# Absorption and Distribution of Toltrazuril and Toltrazuril Sulfone in Plasma, Intestinal Tissues and Content of Piglets after Oral or Intramuscular Administration

**DOI:** 10.3390/molecules26185633

**Published:** 2021-09-16

**Authors:** Hamadi Karembe, Daniel Sperling, Nathalie Varinot, Reynald Magnier, Mathieu Peyrou, Nicolas Guerra, Jiří Smola, Jan Vasek, Barbara Hinney, Anja Joachim

**Affiliations:** 1 Ceva Santé Animale, 10, Avenue de la Ballastière, 33501 Libourne, France; hamadi.karembe@ceva.com (H.K.); daniel.sperling@ceva.com (D.S.); nathalie.varinot@ceva.com (N.V.); reynald.magnier@ceva.com (R.M.); mathieu.peyrou@ceva.com (M.P.); nicolas.guerra@ceva.com (N.G.); 2 Faculty of Veterinary Medicine, University of Veterinary and Pharmaceutical Sciences, 612 42 Brno, Czech Republic; SmolaJ@vfu.cz (J.S.); vasekj@vfu.cz (J.V.); 3 Department of Pathobiology, Institute of Parasitology, University of Veterinary Medicine Vienna, Veterinaerplatz 1, A-1210 Vienna, Austria; Barbara.Hinney@vetmeduni.ac.at

**Keywords:** swine, coccidiosis, *Isospora suis*, prevention, anticoccidials, pharmacokinetics, tissue distribution

## Abstract

Piglet coccidiosis due to *Cystoisospora suis* is a major cause of diarrhea and poor growth worldwide. It can effectively be controlled by application of toltrazuril (TZ), and oral formulations have been licensed for many years. Recently, the first parenteral formulation containing TZ in combination with iron (gleptoferron) was registered in the EU for the prevention of coccidiosis and iron deficiency anemia, conditions in suckling piglets requiring routine preventive measures. This study evaluated the absorption and distribution of TZ and its main metabolite, toltrazuril sulfone (TZ-SO_2_), in blood and intestinal tissues after single oral (20 mg/kg) or single intramuscular (45 mg/piglet) application of TZ. Fifty-six piglets were randomly allocated to the two treatment groups. Animals were sacrificed 1-, 5-, 13-, and 24-days post-treatment and TZ and TZ-SO_2_ levels were determined in blood, jejunal tissue, ileal tissue, and mixed jejunal and ileal content (IC) by high performance liquid chromatography (HPLC). Intramuscular application resulted in significantly higher and more sustained concentrations of both compounds in plasma, intestinal tissue, and IC. Higher concentrations after oral dosing were only observed one day after application of TZ in jejunum and IC. Toltrazuril was quickly metabolized to TZ-SO_2_ with maximum concentrations on day 13 for both applications. Remarkably, TZ and TZ-SO_2_ accumulated in the jejunum, the primary predilection site of *C. suis*, independently of the administration route, which is key to their antiparasitic effect.

## 1. Introduction

Porcine neonatal coccidiosis is a protozoal infection caused by the intestinal parasite *Cystoisospora suis* (previously known as *Isospora suis*) [1]. Cystoisosporosis is a major cause of diarrhea, poor growth performance resulting in significant economic losses in the pig breeding industry. The disease is present worldwide in all types of farrowing facilities and under all types of management systems [1,2]. Subclinical cystoisosporosis is now recognized as an economically important issue as well [3]. Piglets are infected by ingesting sporulated oocysts from contaminated farrowing crates. Upon ingestion of the sporulated oocyst, the infectious sporozoites are released and penetrate the epithelial cells of the ileum and jejunum. Intracellular multiplication (merogony) followed by the development of sexually differentiated cells (gamogony) leads to the destruction of intestinal tissue [4] and disturbances of the gut microbiota [5]. After sexual development and fusion of gametes oocysts are formed, released into the gut lumen, and excreted with the feces. The endogenous development is completed within five to six days. After a temperature-dependent maturation of the oocysts and formation of sporocysts and infectious sporozoites within the oocyst, the life cycle is complete. Disease occurs mostly in the second to third week of life and is characterized by a non-hemorrhagic pasty to liquid diarrhea in the majority of cases. Reduced body weight gain contributes to the economic losses induced by the infection both in clinically affected and subclinically infected piglets [1]. While a number of different drug classes and compounds are effective and registered for the control of coccidiosis in other livestock [6], the only compound that can effectively suppress the endogenous development of *C. suis* is the triazinone toltrazuril (TZ), which is also the only compound registered in the EU for metaphylactic use on affected farms [7]. Anticoccidial triazinones are derivatives of 6-azauracil, an inhibitor of enzymes involved in purine and pyrimidine biosynthesis [8,9]. The first patent applications dealing with the use of new triazinone anticoccidial agents were published between 1974 and 1978 and the first toltrazuril based product was introduced in 1987 [10]. Other triazinones used in other animal species include ponazuril (toltrazuril sulfone; TZ-SO_2_) and diclazuril [6]. Ponazuril is effective against *C. suis* [11] while diclazuril is not [12].

Currently, toltrazuril is used regularly for the metaphylactic control of porcine neonatal cystoisosporosis on affected farms in Europe, Latin America, Canada, and Asian countries [1,13,14]. It largely suppresses oocyst excretion, diarrhea, and complications caused by secondary infections with a single metaphylactic application [12,13,14,15,16,17]. The high anticoccidial activity of toltrazuril is based on its ability to kill asexual and sexual stages of coccidia except the last stage of development, the oocyst. Despite the high efficacy of toltrazuril treatment under laboratory and field conditions, *C. suis* prevalence is still high in important pig production countries in the EU, which may be due to treatment errors, lack of appropriate accompanying hygiene measurements, or drug resistance [18,19]. Information on target tissue concentrations of toltrazuril (in the case of *C. suis* the tissue and luminal content of the small intestines) and elimination times is still missing, although tissue penetration of antiparasitic drugs at the site of infection is a prerequisite for antiparasitic efficacy.

Recently, the first parenteral formulation containing toltrazuril in combination with iron was developed and registered in the EU for the prevention of both coccidiosis and iron deficiency anemia [20,21,22,23].

The route of drug administration affects the systemic bioavailability and the subsequent distribution of compounds. No studies comparing the distribution of toltrazuril and its active metabolite in the intestinal tissues and content after oral and intramuscular application in target species, the pig, has been published thus far. Determining the concentrations of the drugs at the predilection site of the parasite could help to optimize the antiparasitic efficacy [24,25,26].

This study aimed to evaluate and compare the disposition kinetics of toltrazuril and its main metabolite in plasma and in the predilection tissues of *Cystoisospora suis* after oral and intramuscular application in piglets.

## 2. Results

### 2.1. Plasma Concentrations

**Figure 1:**
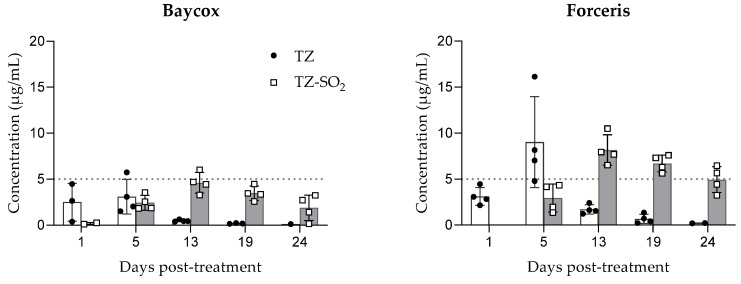
Plasma concentrations of toltrazuril (TZ) and toltrazuril sulfone (TZ-SO_2_) (mean ± SD) following single oral (Baycox 5%) or intramuscular (Forceris) administration to piglets.

Toltrazuril and TZ-SO_2_ were not detectable in any samples collected before treatment. Following oral and intramuscular application, the absorption of toltrazuril was quick with significant plasma concentrations (range: 0.4–4.5 µg/mL) measured at first sampling point, day 1 (24 h) post-treatment. At all sampling times, the plasma TZ concentrations obtained with intramuscular application were higher with maximal plasma concentration (9.03 ± 4.9 µg/mL) observed at 5 days post-treatment. With both routes of application, TZ is quickly metabolized into TZ-SO_2_ with maximal concentrations measured at 13 days post-treatment. At the same time point (13 days post-treatment), the measured TZ concentrations were low, being between 0.4 and 0.6 µg/mL and 1.2 and 2.4 µg/mL for oral and intramuscular routes, respectively (Figure 1).

The metabolization into TZ-SO_2_ started earlier following oral application with measurable plasma concentrations for TZ-SO_2_ at 24 h (day 1) post-treatment (range: 0.11–0.25 µg/mL). Toltrazuril sulfone was slowly eliminated. The concentration measured at the last sampling point, 24 days post-treatment, was 1.88 µg/mL. The metabolization was slightly delayed following intramuscular application, the first measurable concentrations were observed at 5 days post-treatment (second time point of sampling), but the concentrations were higher and more sustained. The concentration measured at last sampling time, 24 days post-treatment, was 4.95 µg/mL (Figure 1).

### 2.2. Tissue and Intestinal Content Concentrations

**Figure 2:**
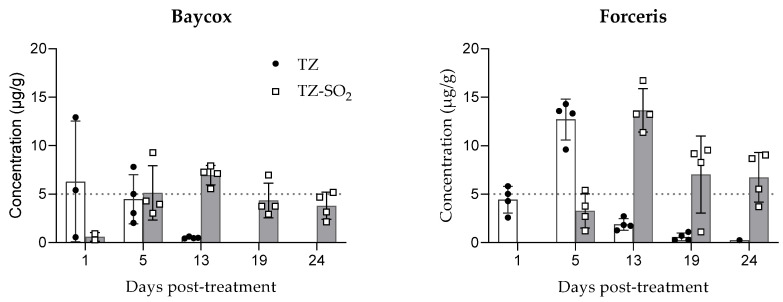
Concentrations of toltrazuril and toltrazuril sulfone (mean ± SD) in jejunal tissue following single oral (Baycox 5%) or intramuscular (Forceris) administration to piglets.

**Figure 3:**
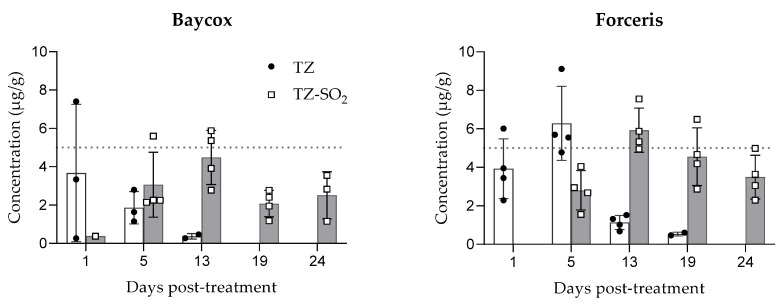
Concentrations of toltrazuril and toltrazuril sulfone (mean ± SD) in the ileum following single oral (Baycox 5%) or intramuscular (Forceris) administration to piglets.

Following oral application, the highest TZ concentrations in intestinal tissues were measured at the first sampling (24 h post treatment). A continuous decrease was observed from day 1 to day 13 post-treatment and the last quantifiable concentrations were observed on days 13–19. Following intramuscular application, the highest TZ concentrations in intestinal tissues were measured at 5 days post-dosing and the last quantifiable concentrations were observed on days 19–24. The concentrations of both TZ and TZ-SO_2_ were higher in the jejunal tissue than in the ileal tissue (Figure 2 and Figure 3).

Metabolization of TZ to TZ-SO_2_ took place earlier with the oral dosing than with the parenteral treatment with first measurable concentrations at 24 h (day 1) post-treatment after oral and at 5 days after intramuscular application. The levels of TZ in intestinal tissues quickly declined after application while TZ-SO_2_ could still be detected on the last day of sampling, 24 days post treatment (Figure 2 and Figure 3).

**Figure 4:**
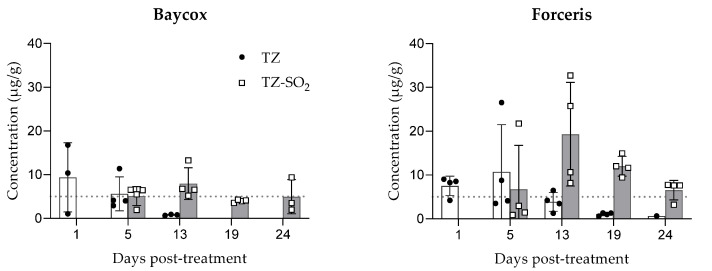
Concentrations of toltrazuril and toltrazuril sulfone (mean ± SD) in the intestinal content following single oral (Baycox 5%) or intramuscular (Forceris) administration to piglets.

The highest TZ concentration in intestinal contents was observed at the first sampling, 24 h (day 1) post treatment, for the oral route (9.39 ± 7.91 µg/g) and on day 5 post treatment for the intramuscular route (10.73 ± 10.80 µg/g). The TZ concentrations decreased after this peak with the last measurable concentrations observed on day 13 for the oral route (<0.50–0.91 µg/g) and days 19 for the intramuscular route (0.62–1.36 µg/g). No TZ-SO_2_ could be measured at the first sampling point (24 h post treatment) for either route of administration. TZ-SO_2_ was first detected on day 5 with highest concentration measured on day 13 for both oral (7.95 ± 3.62) and parenteral (19.32 ± 11.82) administrations (Figure 4).

Overall, the concentrations of TZ and TZ-SO_2_ were higher and more sustained following intramuscular application, and there was a strong correlation between plasma and tissue concentrations of both TZ and TZ-SO_2_ (R^2^ > 0.90).

## 3. Discussion

The development of resistance to the current compounds [27] imposes a continuous search for new compounds with different modes of action. Novel anticoccidial triazinone compounds were recently discovered by using systematic structure-activity relationship studies [28]. The most active compound, nitromezuril, is being developed as an anticoccidial for poultry [29,30,31]. Aminomizuril and ethanamizuril, the active metabolites of nitromezuril, and acetamizuril are also new development candidates [32,33].

The discovery and development of new anticoccidial compounds is a time-consuming process. Meanwhile, repurposing or optimized use of current compounds through new formulations, dosages, and routes of application are considered as means to increase efficacy and delay the development of resistance [34,35,36].

Plasma kinetic data of TZ and TZ-SO_2_ following oral application to piglets is limited [37,38]. Fast absorption and extensive metabolization of TZ to toltrazuril sulfoxide and TZ-SO_2_ were reported following single oral application to piglets [38]. The main metabolite TZ-SO_2_ is slowly eliminated, mainly in the feces [7]. Data were reported as part of registration dossiers for the 5% suspension product for oral administration [7]. Information for different tissues (muscle, fat, skin, liver, and kidney) are available and were obtained as part of residue depletion studies for establishing the withdrawal period of specific products. The characterization of the plasma concentration profiles and results of residue depletion studies of the TZ and TZ-SO_2_ provide understanding of the pharmacological and toxicological features of this anticoccidial compound; however, concentration profiles reached in the tissues of parasite location greatly contributes to improved understanding of its antiparasitic effect in vitro and in vivo.

Recently, the first injectable combination product, Forceris (30 mg toltrazuril/mL; 133.4 mg iron/mL as gleptoferron; Ceva, France) has been developed and registered for the control of piglet coccidiosis and prevention of iron deficiency anemia. Treatment is scheduled from the first to the third day of life (24–96 h after birth) as a single intramuscular injection of a fixed dose of 1.5 mL/piglet corresponding to 45 mg of toltrazuril and 200 mg of iron [37]. Efficacy of Forceris was confirmed in comparison with an established reference product (oral toltrazuril suspension; Baycox 5%, Bayer Animal Health, Leverkusen, Germany) in a *C. suis* challenge model [16,22] as well in a multicenter field trial [20].

Drug concentrations at the predilection site of the parasite are important for its pharmacological effects. *C. suis* is an intracellular parasite that infects enterocytes of the small intestine, mostly of the jejunum and, as infection proceeds, also the ileum. In the jejunal tissue the concentrations of TZ and TZ-SO_2_ were generally higher than those in the ileum after both the oral and the parenteral applications, but the concentration peaks for both metabolites were comparable in the two intestinal compartments—TZ was highest at the beginning of the study period (24 h and 5 days after application) while TZ-SO_2_ concentrations increased until 13 days after application and then decreased again. In both tissues, the parenteral application resulted in higher TZ and TZ-SO_2_ concentrations and a slower decrease of tissue concentrations. Since the time point of infection in relation to treatment cannot be predicted and the endogenous phase of the parasite is limited to the first five days after infection, the “window of opportunity” for intervention against endogenous stages is very small. Consequently, a prolonged effective drug concentration at the target site of infection can be expected to greatly increase the efficacy of anticoccidial drugs. In chickens, the species for which by far the most anticoccidial drugs are developed and used [6], this problem is overcome by applying in-feed treatment for several days during the sensitive phase of life, however, this is not possible in suckling piglets where, for obvious reasons, no in-feed treatment is possible and a single treatment must suffice in order to minimize manipulation and stress of the porcine neonates and to keep a low routine workload [39]. In relation to this, higher and more sustained concentrations of TZ and TZ-SO_2_ were observed following parenteral application, which may provide higher anticoccidial activity under field conditions compared to the oral application when the time point of infection is variable between individual piglets and compound levels may decline to ineffective levels before infections take place. Although the comparison between compliance of intramuscular injection and oral administration of toltrazuril was not the focus of this study, it can be assumed that intramuscular application allows more precise and reliable dosing in the field, since piglets may refuse or regurgitate orally administered products [20], and this may lead to underdosing which in turn promotes the development of antiparasitic resistance.

In conclusion, the higher and more sustained tissue and fecal concentrations after intra-muscular application are likely related to either a mechanism of enterohepatic recirculation, greater intestinal secretion from the blood to the gut lumen, or both. It is assumed that increased and more sustained concentrations of TZ and TZ-SO_2_ at the site of infection with *C. suis* lead to improved efficacy against the parasite, since intracellular stages in the enterocytes of the small intestines constitute the majority of the parasite population during an active infection.

## 4. Materials and Methods

### 4.1. Animals and Treatments

A total of 56 healthy piglets from four litters were enrolled in the study and randomly allocated to two treatment groups by ascending birth weight. Piglets in the first group (group A) received a fixed dose of 45 mg toltrazuril + 200 mg iron as gleptoferron (Forceris, Ceva Santé Animale, Libourne, France) per piglet on the second day of life. Piglets in the second group (group B) received a fixed dose of 200 mg iron/piglet as iron dextran intramuscularly (1 mL Uniferon 200; Pharmacosmos AS, Holbaek, Denmark) on the second day of life and 20 mg/kg of body weight of toltrazuril orally (Baycox 5%, Bayer Animal Health, Leverkusen, Germany) on the fourth day of life (for details on body weights and doses, see Table 1).

Animals (at least four piglets per time point and treatment group) were sacrificed 1-, 5-, 13-, or 24-days post treatment. At the end of the animal phase, all remaining animals were sacrificed and sampled. From each piglet, the following samples were collected: blood, jejunal tissue, ileal tissue, jejunal content, and ileal content.

### 4.2. Blood and Tissue Samplings and Analytical Procedures

#### 4.2.1. Blood Samplings and Sample Preparation for HPLC

Blood samples were collected into heparinized tubes and stored immediately at 4 °C until centrifugation. Samples were centrifuged (2500× *g*, 10 min, +4 °C) and the resulting plasma was collected and distributed into two dry polypropylene tubes of 3.5 mL.

To a 150 μL aliquot of each plasma sample, 15 μL of internal standard (IS) solution (100 µg/mL fusidic acid in acetonitrile) and 500 μL of water were added and vortexed. After the addition of 4500 µL of tert-butyl methyl ether, the sample tubes were capped, vortexed for 5 min, and then centrifuged for 5 min at 2000× *g*. After freezing of the aqueous phase in a dry-ice bath, the organic phase was transferred to a glass tube, evaporated to dryness at 30 °C and reconstituted with 300 µL of the mixture acetonitrile/water (50/50, *v*/*v*).

#### 4.2.2. HPLC Analysis of Blood Serum

Plasma concentrations of toltrazuril and its main sulfone metabolite were determined using a validated HPLC-UV method [40]. Chromatography was performed on an Agilent 1200 LC system (Agilent Technologies, Les Ulis, France). The separation was achieved on a C18 Kinetec column 2.6 µm, 4.6 × 100 mm (Phenomenex, Le Pecq, France). The UV detector was set at 248 nm. An isocratic mobile phase of 5 mM ammonium acetate containing 1% of acetic acid: acetonitrile (52:48, *v*/*v*) was applied at a flow rate of 1 mL/min. The injection volume was 60 μL. The temperatures of the column and autosampler were kept at 40 °C and 4 °C, respectively. The retention times of the compounds were about 4.0, 7.5, and 11.0 min for toltrazuril-sulfone, toltrazuril, and fusidic acid, respectively. The method was linear in the concentration range of 0.1–25 µg/mL. The intra- and inter-assay precision (% CV) values were within 5.83 and 6.00% for toltrazuril and toltrazuril-sulfone, respectively. The inter assay accuracy ranged from 5.00% to 10.85% for toltrazuril and from 0.00% to 6.37% for toltrazuril sulfone. The limit of quantification (LOQ) of toltrazuril and toltrazuril sulfone in the plasma was established at 0.1 µg/mL.

#### 4.2.3. Tissue and Intestinal Content Sampling and Sample Preparation for HPLC Analysis

Necropsy and tissue sample collection took place at 1, 5, 13, and 24 days after treatment. From each piglet, the following tissue samples were collected: two pieces of jejunum and two pieces of ileum were collected (ca. 10 cm/piece) as well as contents from ileum and jejunum (ca. 10 mL/sample). Intestines were opened lengthwise and intestinal content was collected by gentle scraping. Contents of the corresponding parts of the jejunum and ileum were separated and equal amounts (5 mL each) were mixed in order to obtain a final sample representing the total content. The collected samples were stored at −80 °C before analysis.

A total of 20 µL of internal standard was added to 1 g of intestinal tissue or intestinal contents previously homogenized. After mixing for 10 s, 3 mL of ethyl acetate were added to each tissue samples. The sample tubes were capped, vortexed for 10 min, and then centrifuged for 5 min at 2000× *g*. Using a disposable pipet, the supernatant was transferred to a glass tube. An additional 3 mL ethyl acetate was added to the pellet containing the extracted tissue, and then vortexed and centrifuged as before. The resulting supernatant was transferred to the tube containing the supernatant from the first extraction. Sample tubes were evaporated to dryness at 45 °C using N_2_. Furthermore, 1 mL of acetonitrile was added to each tube, vortexed for 10 min, and centrifuged at 3000× *g* for 10 min. The supernatant was transferred to a glass tube, evaporated to dryness at 45 °C using nitrogen (N_2_) and reconstituted with 300 µL of the mixture H_3_PO_4_ 0.085% in water-acetonitrile (50/50, *v*/*v*). Sample extracts were filtered through a 13 mm/0.45 μm GHP Acrodisk filter (VWR international SAS, Fontenay-sous-Bois, France) and stored at 4 °C before injection.

#### 4.2.4. HPLC Analysis of Tissue and Intestinal Content Samples

Tissue concentrations of toltrazuril and its main sulfone metabolite were determined using a validated HPLC-UV method. Chromatography was performed on an Agilent 1200 LC system (Agilent Technologies, Les Ulis, France). The separation was achieved on a C18 Kinetec column 2.6 µm, 3 × 100 mm (Phenomenex, Le Pecq, France). The UV detector was set at 248 nm. The column temperature was maintained at 25 °C as mobile phase, H_3_PO_4_ (0.085%) in water (solvent A) and acetonitrile (solvent B) were used. The gradient elution program was 40% solvent B as initial conditions. This composition was maintained for 4 min, followed by a linear gradient up to 45% in 5 min, followed by a linear gradient up to 50% in 5 min, followed by a linear gradient up to 70% in 2 min, followed by a linear gradient up to 95% in 1 min before returning to the initial conditions for 1 min. The retention times of the compounds were about 10.8 min and 16.1 min for toltrazuril-sulfone and toltrazuril, respectively. The method was linear in the concentration range of 250–10,000 µg/kg. The intra-assay precision, inter-assay precision, and accuracy are summarized in Table 2.

The limit of quantification (LOQ) of toltrazuril and toltrazuril sulfone was 0.250 µg/g for jejunum and ileum and 0.500 µg/g for the intestinal content.

##### Statistical Analysis

Statistical analysis and graphing were undertaken using GraphPad Prism 8 for Windows (GraphPad Software, LLC, San Diego, CA, USA). All data are represented as arithmetic mean ± standard deviation (SD) in the tables and figures. The low samples size excluded the use of comparison tests.

##### Ethical Statement

Animal experimentation was approved by the Ethics Committees of the Faculty of Veterinary Medicine of the University of Veterinary and Pharmaceutical Sciences, Brno (case No 42-2017), the University of Veterinary Medicine Vienna and the Austrian national authorities according to § 26ff of Animal Experiments Act, Tierversuchsgesetz 2012-TVG 2012 (license number: BMWF-68.205/0034-WF/V/3b/2016; Austrian Federal Ministry of Science, Health and Economy).

## Figures and Tables

**Table 1:** Body weights on the day of treatment and treatment doses of the two treatment groups.

Group	N	Mean Body Weight (Standard Deviation)		
		SD 2	SD 4	Toltrazuril Dose
A—Forceris Parenteral treatment with toltrazuril on SD 2	29	1.57 (0.35) Min: 0.97 Max: 2.54	1.84 (0.38) Min: 1.25 Max: 2.97	45 mg/piglet Mean = 29.0 mg/kg Min: 17.7 mg/kg Max: 46.6 mg/kg
B—Baycox Oral treatment with toltrazuril on SD 4	27	1.57 (0.30) Min: 0.89 Max: 2.19	1.87 (0.35) Min: 1.21 Max: 2.19	20 mg/kg

**Table 2:** The intra-assay precision (%CV), inter-assay precision (%CV), and accuracy (%dev) of the HPLC analysis.

Matrix	Parameter	Analyte	
		Toltrazuril	Toltrazuril Sulfone
Content	%CV	≤1.80	≤13.87
	%CV	≤2.80	≤5.07
	%dev	−2.67 to 0.48	−1.29 to 7.18
Ileum or jejunum	%CV	≤3.09	≤3.45
	%CV	≤3.45	≤3.90
	%dev	−3.26 to 0.54	−3.79 to 0.29

## Data Availability

All data are included in this publication.

## Sample Availability

Samples have been consumed during analyses and are thus not available.
